# Halotolerant endophytic bacteria alleviate salinity stress in rice (*oryza sativa* L.) by modulating ion content, endogenous hormones, the antioxidant system and gene expression

**DOI:** 10.1186/s12870-023-04517-z

**Published:** 2023-10-14

**Authors:** Saleem Asif, Rahmatullah Jan, Nari Kim, Sajjad Asaf, Lubna, Muhammad Aaqil Khan, Eun-Gyeong Kim, Yoon-Hee Jang, Dibya Bhatta, In-Jung Lee, Kyung-Min Kim

**Affiliations:** 1 Department of Applied Biosciences, Graduate School, Kyungpook National University, Daegu, 41566 South Korea; 2 Coastal Agriculture Research Institute, Kyungpook National University, Daegu, South Korea; 3 Natural and Medical Science Research Center, University of Nizwa, Nizwa, Oman; 4 Department of chemical and life sciences, Qurtuba university of science and information technology, Peshawar, Pakistan

**Keywords:** Rice (*Oryza sativa* L.), Salinity stress, Hormones, Endophytes, Antioxidants, Minerals

## Abstract

Excessive salinity reduces crop production and negatively impacts agriculture worldwide. We previously isolated endophytic bacterial strains from two halophytic species: *Artemisia princeps* and *Chenopodium ficifolium*. We used three bacterial isolates: ART-1 (*Lysinibacillus fusiformis*), ART-10 (*Lysinibacillus sphaericus*), and CAL-8 (*Brevibacterium pityocampae*) to alleviate the impact of salinity stress on rice. The impact of 160 mM NaCl salinity on rice was significantly mitigated following inoculation with these bacterial strains, resulting in increased growth and chlorophyll content. Furthermore, *OsNHX1*, *OsAPX1*, *OsPIN1* and *OsCATA* expression was increased, but *OsSOS* expression was decreased. Inductively coupled plasma mass spectrometry (ICP-MS) revealed reduced K^+^ and Na^+^ levels in shoots of bacteria-inoculated plants, whereas that of Mg^2+^ was increased. Bacterial inoculation reduced the content of total flavonoids in rice leaves. Salinized plants inoculated with bacteria showed reduced levels of endogenous salicylic acid (SA) and abscisic acid (ABA) but increased levels of jasmonic acid (JA). In conclusion, the bacterial isolates ART-1, ART-10, and CAL-8 alleviated the adverse effect of salinity on rice growth, which justifies their use as an eco-friendly agricultural practice.

## Introduction

Soil salinity is an extensive abiotic stress that drastically affects crop production and food security worldwide [1, 2]. Excessive salinity currently affects 20% of irrigated lands (about 62 million hectares) and is expected to affect over 50% of arable lands by 2050 [3, 4]. The primary contributor to the problem of salinity in agricultural lands is the buildup of salts (particularly sodium and chloride ions) that disturbs water balance of the plant, upsets nutrient balance, and causes ion toxicity; which eventually interferes with various physiological processes, resulting in chlorosis and necrosis [1, 5]. The soil becomes saline, when electrical conductivity of the saturated extract exceeds 4 dS m^− 1^ [6]. Plants under salinity stress experience several morphological and developmental changes, including poor seed germination and seedling growth, low yield that can be related to physiological and molecular changes [7]. Salinity accelerates the production of reactive oxygen species (ROS) in the plant body that can damage lipids, nucleic acids, and cell membranes; despite their role in programmed cell death at low doses under normal circumstances [2, 8].

Salt stress can affect seed germination, seedling growth, tiller and spikelet number, and plant yield [9]. It disturbs water uptake leading to osmotic stress and loss of turgor [10]. High salinity reduces chlorophyll content of leaves, and the magnitude of reduction depends on genotype and plant developmental stage [11, 12]. In response to salinity, plants activate their antioxidant defense system [superoxide dismutase (SOD), DPPH scavenging activity, phenolics, and flavonoids ; which can aid in amelioration of the impact of salinity by controlling the biosynthesis of ROS [2, 8, 13].

The response of plants to salinity stress depends on their developmental stage; it involves gene expression as well as regulation of hormones and signaling pathways [4, 14]. Gene regulation is crucial for plants that can adapt to unfavorable conditions [4]. The rice genome contains five *OsNHX1* genes and three genes of the *SOS1* family that play critical roles in response to salinity stress [6, 15]. *OsNHX1* is an important Na^+^/H^+^ antiporter in rice and plays a crucial role in salinity and drought tolerance [2]. Both *SOS3* and *SOS2* form a complex and activate the antiporter *SOS1*, which plays the most prominent role among the members of *SOS* family in response to salinity stress via exclusion of Na^+^ from cells [16]. Phytohormones are low-molecular weight chemical signals that play crucial roles in plant growth and development. Several auxin influx genes (such as *PIN* and *YUCCA*) are very helpful in plant salt tolerance via their essential role in auxin biosynthesis [2]. Abscisic acid (ABA), salicylic acid (SA), and jasmonic acid (JA) are the most prominent hormones involved in resistance to abiotic stress. Abscisic acid and SA exhibit antagonistic behavior in response to abiotic stress [17]. Abscisic acid is synthesized in the roots; it is a first-line defense mechanism that mitigates salinity stress through inducing stomatal closure, adaptive physiological responses and regulation of gene expression [2, 18]. Similarly, SA is an endogenous hormone that counteracts abiotic stress by enhancement of antioxidant systems, synthesis of osmolytes and promotion of photosynthesis under stress conditions [2, 19]. Jasmonic acid is a plant growth-promoting hormone that plays a key role in stress resistance [20]; it can perform this role via modulating ABA balance in rice [21].

Several strategies, such as molecular-assisted breeding and plant genetic engineering [22], have been used to develop salinity-resistant crops. However, these techniques are inefficient, tedious, and time-consuming [1]. Alternative approaches for promoting sustainable agriculture [such as manipulation of plant growth-promoting bacteria (PGPB)] are gaining importance to mitigate the impact of salinity on crop plants [8]. These PGPB adopt various direct and indirect mechanisms such as production of indole-3-acetic acid (IAA), exopolysaccharides (EPS) and organic acid siderophores in addition to their ability for phosphate solubilization [23]. The role of PGPB in alleviation of the impact of salinity stress has been documented in rice [23], tomato [24], cucumber [25], and maize [26].Rice is one of the most dominant staple foods for over half of the world’s population. It is considered a salt-sensitive plant in the seedling, panicle formation, flowering, and pollination stages [27, 28]. Manipulation of PGPB, such as *Acinetobacter, Azotobacter*, *Bacillus* sp., *Serratia* sp., *Pseudomonas* sp., and *Rhizobium* sp., to enhance plant growth and crop yield under the impact of salinity is well-documented [2, 23]. In an earlier work, we screened several bacterial isolates of plant growth promoting potentialities; three of which (ART-1, ART-10, and CAL-8) were inoculated into rice plants to evaluate their role in improvement of rice growth and alleviation of the impact of salinity stress. The participation of these bacterial isolates in alleviation of salinity-induced oxidative stress, maintenance of ionic and hormonal homeostasis under salt stress and modulation of expression of salinity-related genes have been evaluated.

## Results

### Isolation, screening, and identification of bacterial isolates

**Fig. 1:**
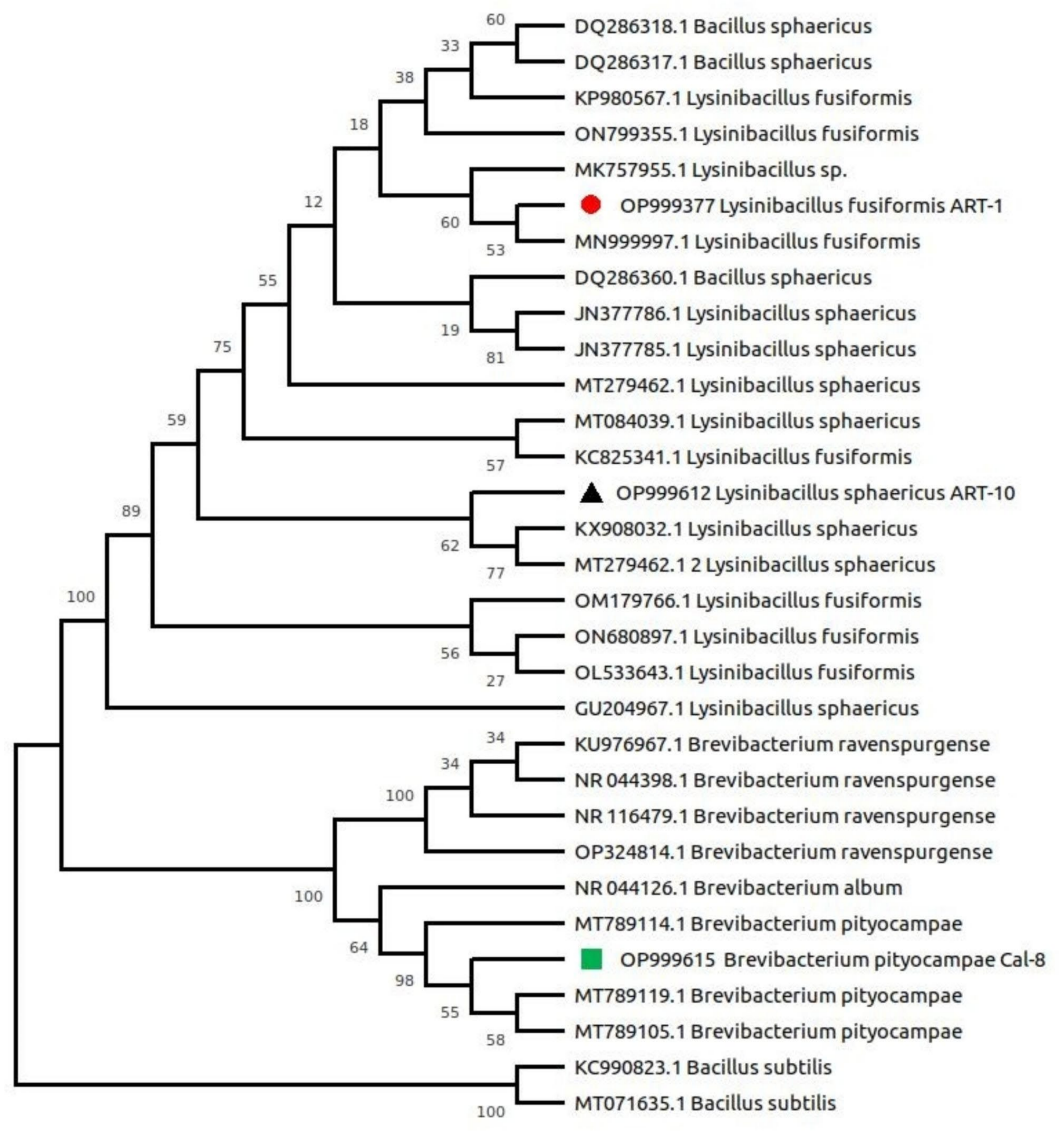
Phylogenetic tree of ART-1, ART-10, and CAL-8 bacterial isolates based on the sequence obtained from 27 F and 1492R primers of 16 S rDNA. Each node indicates the percentage of confidence levels generated from 1000 bootstrap trees

The endophytic bacterial strains ART-1, ART-10 and CAL-8 were isolated from the roots of *Artemisia princeps* and *Chenopodium ficifolium* from Pohang Beach and identified by phylogenetic analysis (Fig. 1). The three isolates have high 16 S rRNA sequence similarity with *Lysinibacillus fusiformis*, *Lysinibacillus sphaericus*, and *Brevibacterium pityocampae*, respectively. The phylogenetic analysis reveals that ART-1 form a clade with *L. fusiformis* (MN999997) and *Lysinibacillus* sp. (MK757955.1). Similarly, ART-10 shared clade of high bootstrap values with *L. sphaericus* (KX908032.1) and *L. sphaericus* (MT279462.1). On the other hand, CAL-8 shared a clade with *B. pityocampae* (MT789119.1). These sequence data have been submitted to the NCBI GenBank under accession numbers OP999377, OP999612, and OP999615 for ART-1, ART-10, and CAL-8, respectively.

### Effect of bacterial inoculation on rice growth under salinity stress

**Fig. 2:**
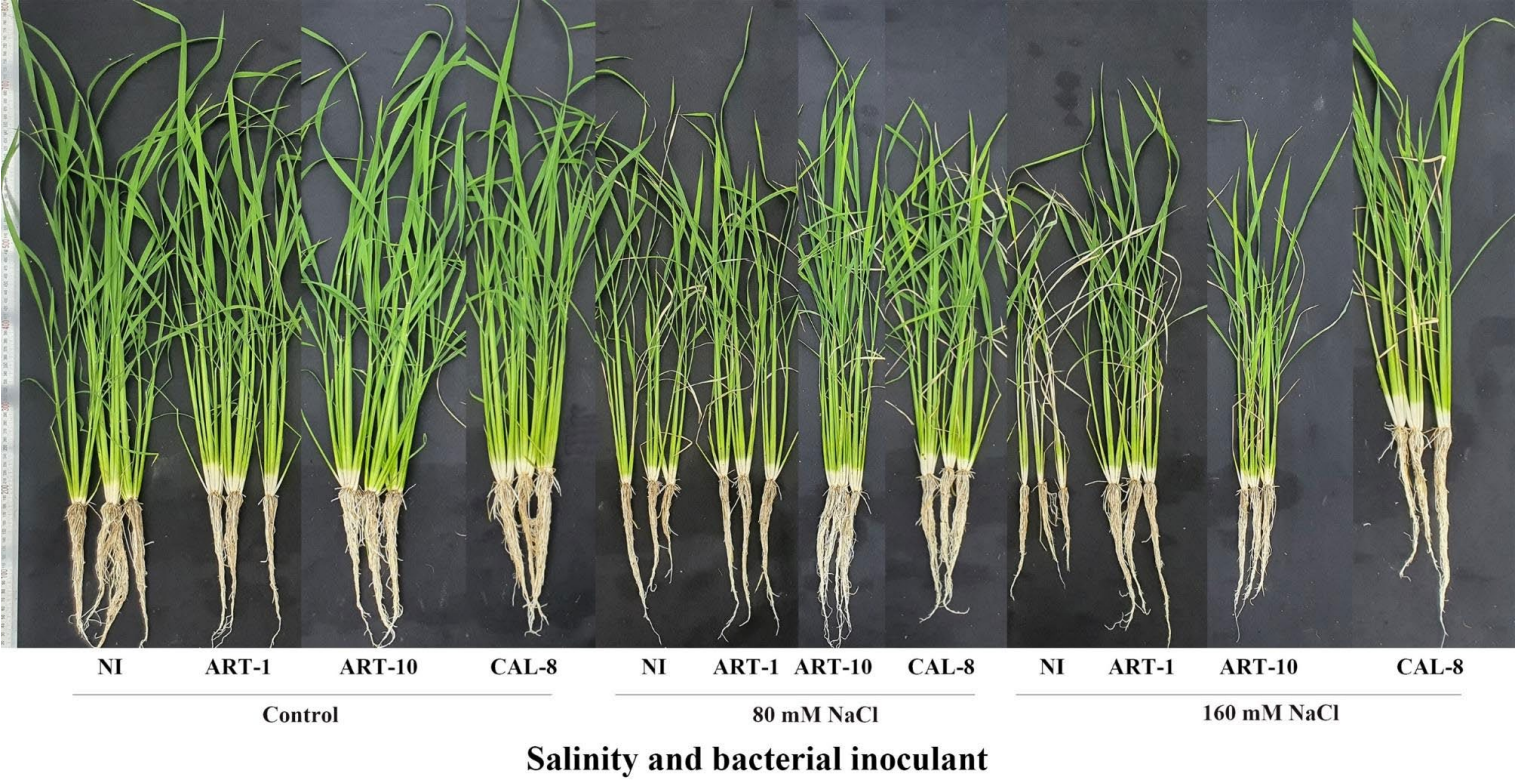
Effect of ART-1, ART10, and CAL-8 bacterial isolates on growth of rice under the impact of 80 and 160 mM NaCl salinity compared with the non-salinized control

**Fig. 3:**
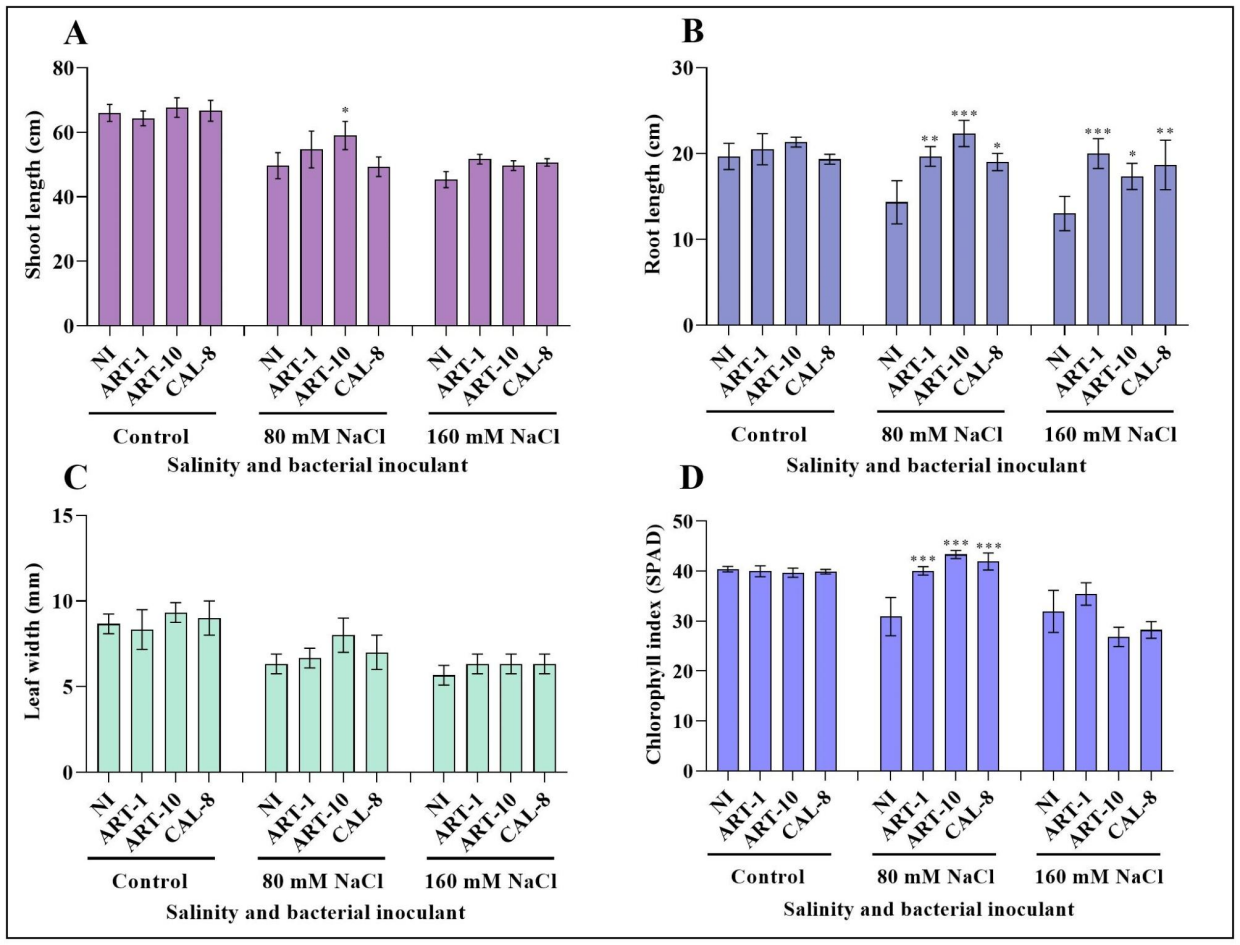
Effect of ART-1, ART-10, and CAL-8 bacterial isolates on growth of rice under salinity stress. Each column represents the mean of three replicates ± SE. columns with the no letter are non-significantly different at P < 0.05. Inoculated plants are compared with non-inoculated plants

Salinity stress negatively affected rice growth (Fig. 2**).** The reduction in shoot length, root length, leaf width and chlorophyll content under the impact of 160 mM NaCl amounted to 31%, 33%, 33% and 20%, respectively below the control in non-inoculated plants. However, the application of bacterial isolates counteracted the effect of salinity and caused a significant increase in plant growth. Inoculation with ART-1 increased shoot length, root length, leaf width and chlorophyll content of the 160 mM NaCl-stressed plants by 13%, 53%, 1% and 10%, respectively; meanwhile, the increases due to ART-10 amounted to 9%, 33%, 20% and 15%, respectively and those due to CAL-8 amounted to 11%, 43%, 40% and 11%, respectively. However, the beneficial effect of bacterial inoculation on rice growth was non-significant in absence of salt stress (Fig. 3**)**.

### Relative gene expression

**Fig. 4:**
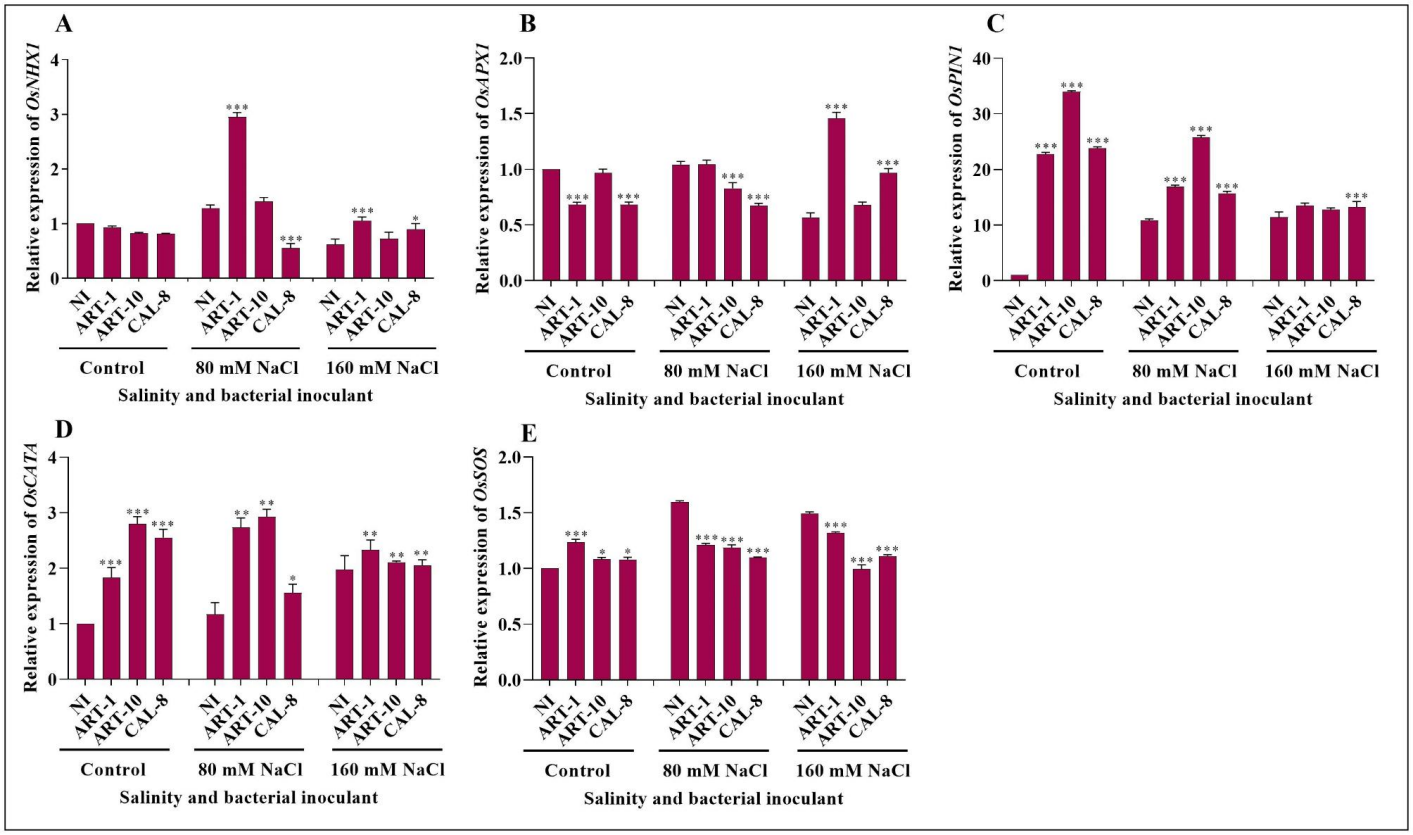
Effect of ART-1, ART-10, and CAL-8 bacterial isolates on relative gene expression of rice under salinity stress. **(A)***OsNHX1*, **(B)***OsAPX1*, **(C)***OsPIN1*, **(D)***OsCATA*, and **(E)***OsSOS*. Actin was used as the reference gene. Each column represents the mean of three replicates ± SE. Columns with the no letter are non-significantly different at P < 0.05. and columns indicate a significant difference (* p < 0.05, ** p < 0.01, *** p < 0.001), according to two-way ANOVA and the Bonferroni post hoc test. Inoculated plants are compared with non-inoculated plants

The relative expression of some selected genes of rice in response to salinity stress and bacterial inoculation was monitored using qRT-PCR (Fig. 4). In absence of salinity, bacterial inoculation led to non-significant reduction in *OsNHX1* expression. But, *OsNHX1* expression was significantly upregulated by 80 mM NaCl, and under the impact of this moderate salinity gene expression was highly significantly upregulated by inoculation with ART-1 and ART-10, while a highly significant reduction was observed in CAL-8- inoculated plants (Fig. 4A**)**. Generally, 160 mM NaCl led to significant reduction in *OsNHX1* expression, with mild effect of bacterial inoculation under the impact of this high salinity. (Fig. 4A**)**. In non-salinized plants, *OsAPX1* expression was significantly downregulated by ART-1 and CAL-8 inoculation with non-significant effect of ART-10 (Fig. 4B**)**. Under the impact of 80 mM NaCl-, significant downregulation of *OsAPX1* expression was found in ART-10- and CAL-8-inoculated plants versus non-significant effect of ART-1. While 160 mM NaCl, either alone or in combination with ART-10 led to significant downregulation of *OsAPX1* expression, inoculation of 160 mM NaCl-stressed plants with ART-1 led to highly significant upregulation of gene expression versus non-significant effect of CAL-8 (Fig. 4B**)**. In absence of salinity, *OsPIN1* expression was radically upregulated by bacterial inoculation, particularly ART-10 (Fig. 4C**)**. Both salinity and bacterial inoculation led to highly significant upregulation of *OsPIN1* expression. Generally, the effect of bacterial inoculation in upregulating the expression of *OsPIN1* was most evident in absence of salinity and diminished with the increase in salinity level (Fig. 4C**)**. *OsCATA* expression was significantly upregulated by salinity in non-inoculated and ART-1-inoculated plants, but the reverse was true in ART-1- and CAL-8- inoculated plants. In non-salinized and moderately salinized (80 mM NaCl) plants, bacterial inoculation. with the exception of CAL-8-inoculated plants, significantly upregulated *OsCATA* expression. However, under the impact of high salinity (160 mM NaCl) the effect of bacterial inoculation in upregulating *OsCATA* expression was limited and comparable among the different bacterial isolates. (Fig. 4D**)**. In absence of salinity, *OsSOS* expression was moderately upregulated by bacterial inoculation; but, gene expression was marginally affected by salinity and was significantly downregulated by bacterial inoculation in the salinized plants (Fig. 4E**)**.

### Mineral content

**Fig. 5:**
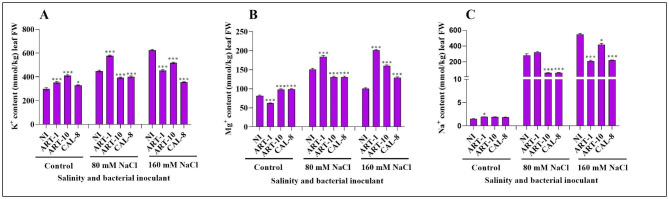
Effects of isolates ART-1, ART-10, and CAL-8 bacterial isolates on K^+^, Mg^2+^, and Na^+^ contents of rice leaves under salinity stress. **(A)** K^+^ content, **(B)** Mg^2+^ content, and **(C)** Na^+^ content. Each column represents the mean of three replicates ± SE. columns with the no letter are non-significantly different at P < 0.05 and asterisks denote a significant difference (* p < 0.05, ** p < 0.01, *** p < 0.001), according to the Bonferroni post hoc test

In non-inoculated plants, K^+^ content of rice leaves was considerably increased under the impact of salinity, where the increase amounted to 52% and 112% under 80 mM NaCl and 160 mM NaCl, respectively above the control plants. But such salinity-dependent increase in leaf K^+^ content was less evident in inoculated plants. Whereas bacterial inoculation led to significant increases in leaf K^+^ content of control plants, the reverse was true in salinized plants with the exception of ART-1 inoculation of moderately salinized (80 mM NaCl) plants where a significant increase was found (Fig. 5A). Mg^2+^ content of rice leaves was generally increased under the impact of salinity, which also modified the effect of bacterial inoculation. In control and highly salinized plants, bacterial inoculation significantly increased Mg^2+^ content of rice leaves, with mild effect under the impact of 80 mM NaCl. Nevertheless, ART-1 represented an exception where it led to a significant decrease in leaf Mg^2+^ content in control plants versus marked increases in the salinized plants (Fig. 5B). Expectedly, Na^+^ content of rice leaves was progressively and radically increased with the increase in salinity. By contrast, bacterial inoculation significantly reduced Na^+^ content of leaves of the salinized plants with non-significant effect in control plants (Fig. 5C).

### Histochemical analysis

**Fig. 6:**
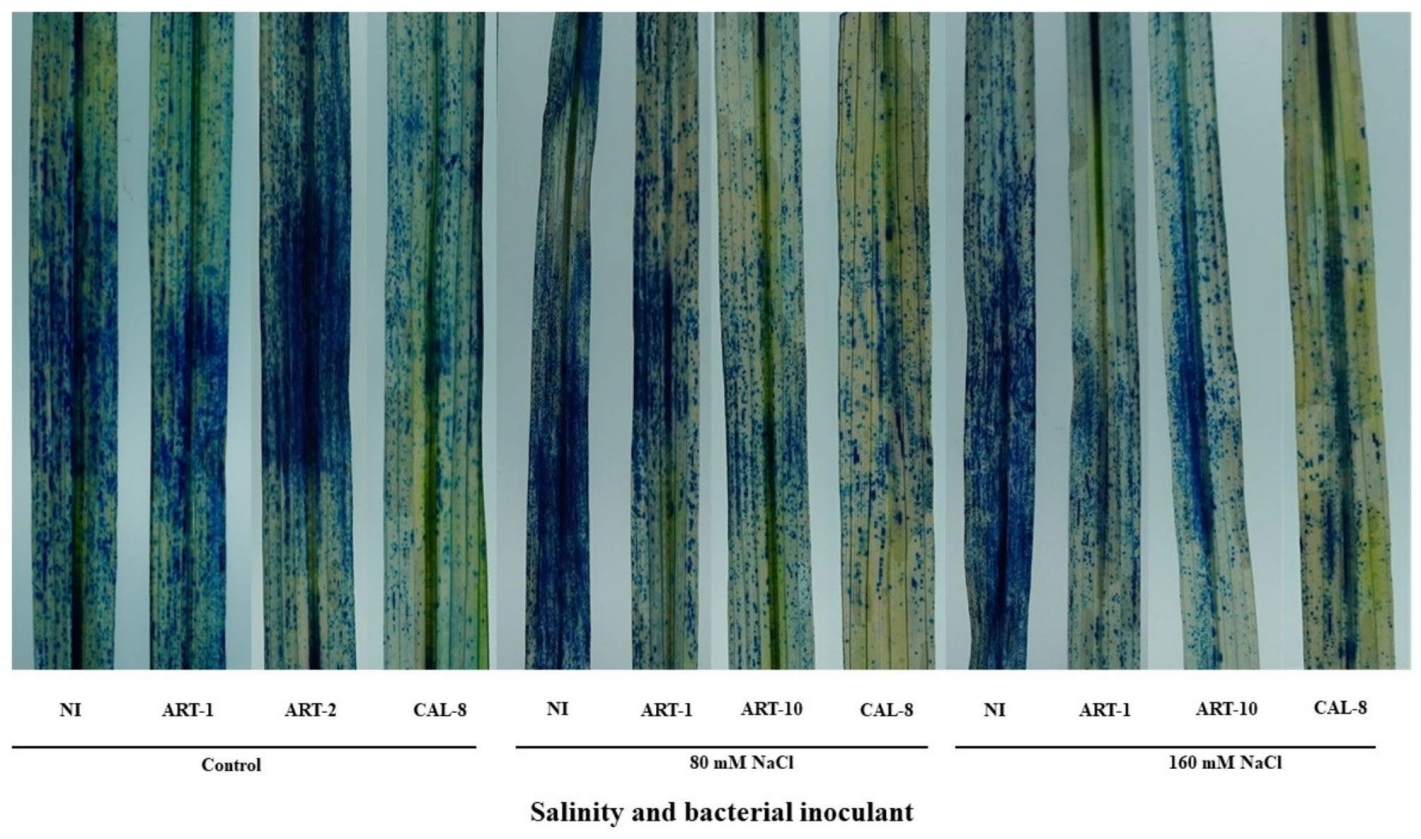
Effects of different bacterial isolates under salinity stress through induction of the hypersensitive response

Cell death was evaluated by using trypan blue staining. Cell death was observed in NaCl-stressed plants at 80 mM and 160 mM. Inoculation with different bacterial isolates reduced the occurrence of cell death under salt stress (Fig. 6).

### Regulation of enzymatic and non-enzymatic antioxidants under salinity stress

**Fig. 7:**
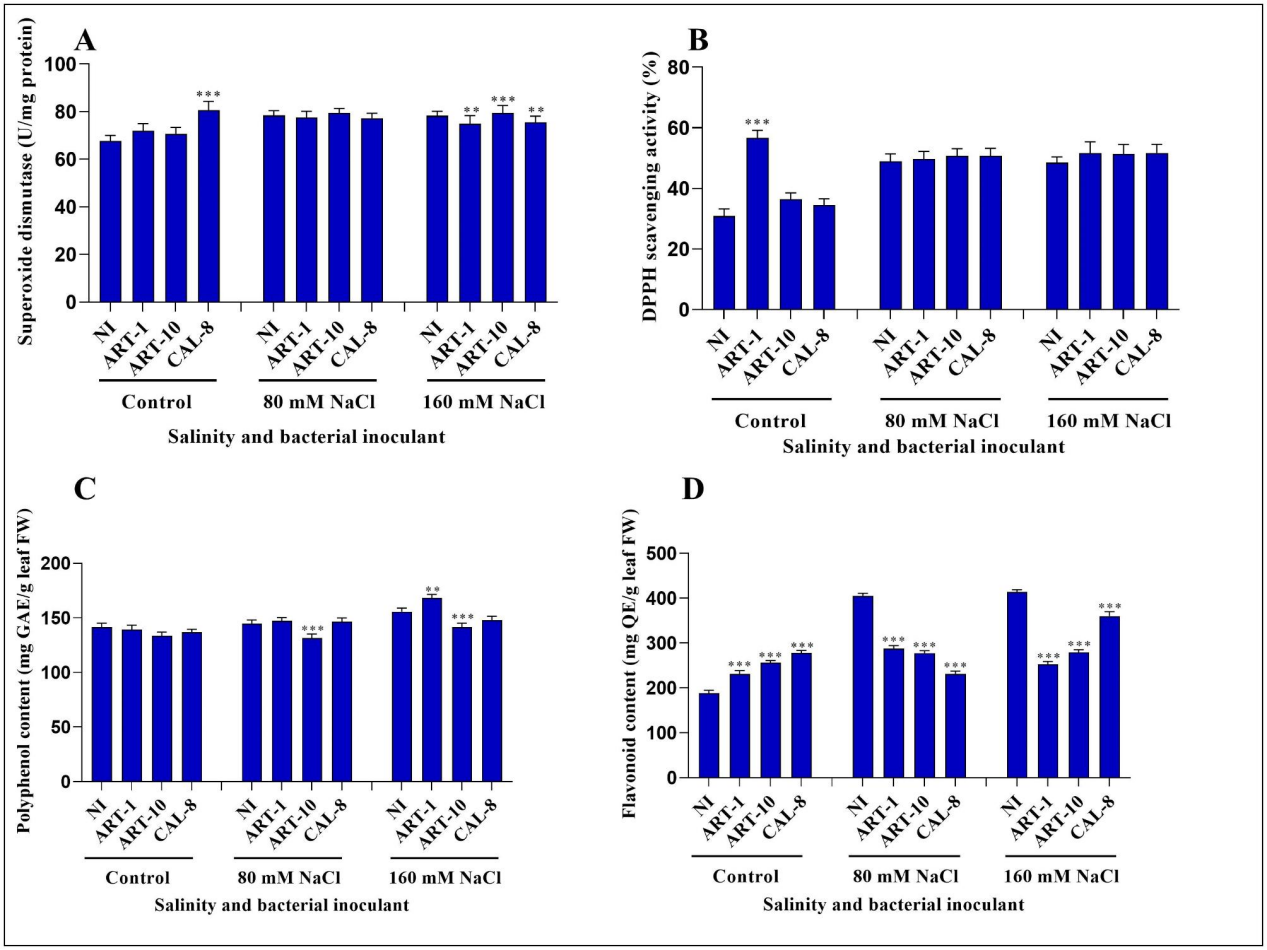
Effects of bacterial isolates ART-1, ART-10, and CAL-8 on different antioxidants under salinity stress. **(A)** SOD, **(B)** DPPH, **(C)** total phenolic content (TPC), and **(D)** total flavonoid content (TFC). Each column represents the mean of three replicates ± SE. columns with the no letter are non-significantly different at P < 0.05 and asterisks denote a significant difference (* p < 0.05, ** p < 0.01, *** p < 0.001), according to the Bonferroni post hoc test

The activities of different antioxidants that is (SOD, DPPH scavenging activity, total phenolic content (TPC), and total flavonoid content (TFC)) in response to salinity and bacterial inoculation were monitored. In general, the activities of SOD and DPPH as well as the contents of TPC, and TFC were increased to different extents in rice leaves under salinity stress, with varying effect of bacterial inoculation. The increases in SOD activity due to salinity and bacterial inoculation were mild (Fig. 7A). By contrast, DPPH scavenging activity exhibited marked salinity-induced increase but with mild effect of bacterial inoculation except the pronounced increase due to ART-1 in control plants (Fig. 7B). The content of phenolics in rice leaves was subjected to mild increases in response to salinity versus mild decreases due to bacterial inoculation (Fig. 7C). On the other hand, the effect of treatments on flavonoid content of leaves was pronounced with marked interaction. The salinity-induced increase in flavonoid content was more evident in non-inoculated relative to inoculated plants. Likewise, while bacterial inoculation increased flavonoid content of leaves in control plants, the reverse was true under the impact of salinity (Fig. 7D).

### Regulation of endogenous phytohormones under salinity stress

**Fig. 8:**
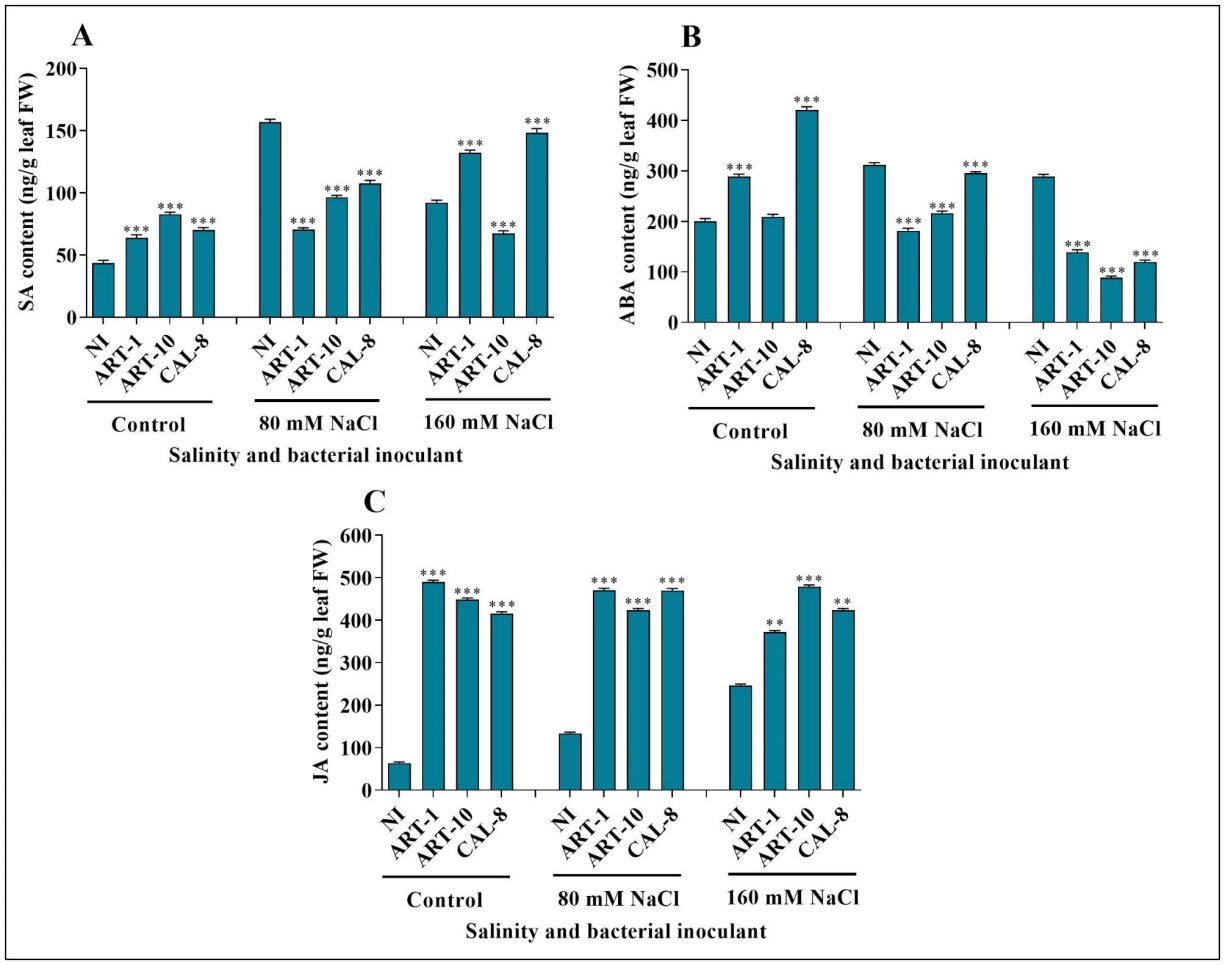
Effects of isolates ART-1, ART-10, and CAL-8 on different endogenous hormones under salinity stress. **(A)** SA, **(B)** ABA, and **(C)** JA. Each column represents the mean of three replicates ± SE. columns with the no letter are non-significantly different at P < 0.05 and asterisks denote a significant difference (* p < 0.05, ** p < 0.01, *** p < 0.001), according to two-way ANOVA analysis and the Bonferroni post hoc test. Inoculated plants are compared with non-inoculated plants. The experiments were performed in triplicate

The application of different bacterial isolates–with the exception of ART-10 at 160 mM NaCl– increased endogenous SA content of rice leaves in the control and highly salinized (160 mM NaCl) plants but reduced it in the moderately salinized (80 mM NaCl) plants. Salinity generally increased SA content of rice leaves with peaking at 80 mM NaCl for non-inoculated and ART-10-inoculated plants but with a progressive increase with the increase in salinity up to 160 mM NaCl for plants inoculated with ART-1 and CAL-8. (Fig. 8A). Similarly, bacterial inoculation significantly increased endogenous ABA levels in rice leaves in the control plants but reduced it in the highly salinized plants with inconsistent effect at moderate salinity. Salinity stress increased endogenous ABA levels I rice leaves of the control plants; but in the inoculated plants, salinity led to marked reductions in ABA content, and the reduction was most severe in the CAL-8-inoculated plants (Fig. 8B). Endogenous JA content was higher in bacterial-inoculated plants than in non-inoculated plants, and the effect was most pronounced in absence of salinity. The effect of salinity on JA content of rice leaves varied according to bacterial inoculation, where salinity increased it in the non-inoculated plants versus a decreasing effect in the ART-1-inoculated plants and a mild effect in the ART-10- and CAL-8-inoculated plants (Fig. 8C).

### Confirmation of bacterial isolates in inoculated plants

The 16 S gene sequencing result showed that ART-1, ART-10 and CAL-8 were present in respective inoculated plants while no bacterial strains were present in non-inoculated plants. The sequence results showed that three isolates have high 16 S rRNA sequence similarity (99–100%) with *Lysinibacillus fusiformis*, *Lysinibacillus sphaericus*, and *Brevibacterium pityocampae*, respectively. The sequence data has been already submitted to the NCBI GenBank under accession numbers OP999377, OP999612, and OP999615 for ART-1, ART-10, and CAL-8, respectively that’s why we didn’t submit again.

### Correlation among plant growth, hormonal content, antioxidant and gene expression

**Fig. 9:**
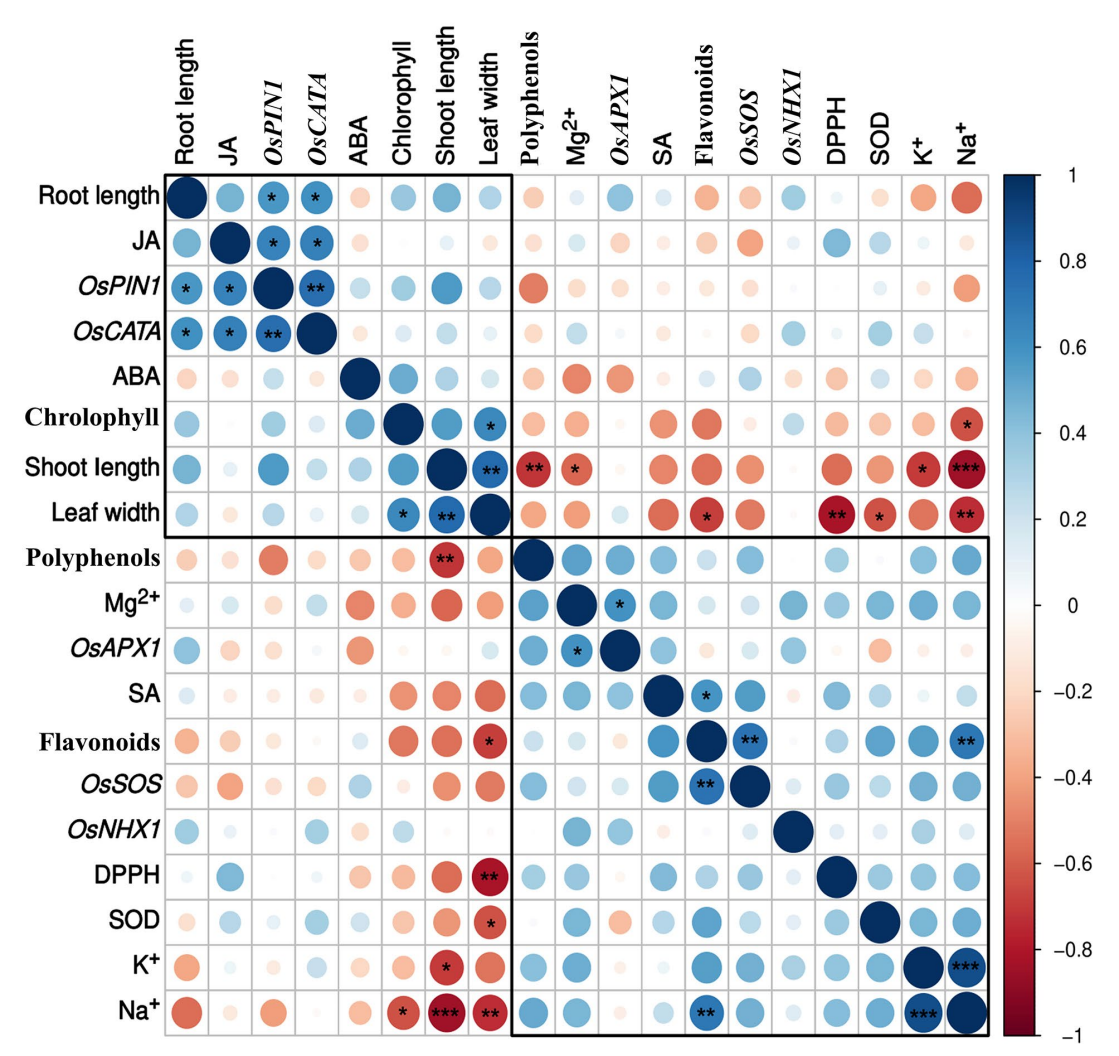
Pearson’s correlation matrix between rice growth attributes, hormonal content, antioxidants, ion content and gene expression in salt-stressed inoculated and non-inoculated plants. Correlations are displayed in blue (positive) and red (negative); color intensity and circle size are proportional to the correlation coefficient. Asterisks denote a significant difference (* p < 0.05, ** p < 0.01, *** p < 0.001)

In the current study we investigated the correlations between various growth attributes, hormonal content, antioxidant activity and gene expression in rice plants. Pearson correlation analysis was performed to examine the relationship between the parameters **(**Fig. 9**).** Our results indicate a positive correlation between shoot length, root length, leaf width, and chlorophyll index, indicating that these growth attributes are interdependent. Interestingly, these growth attributes showed a negative correlation with Na^+^, salicylic acid (SA), flavonoid and polyphenol contents. This suggests that the accumulation of these metabolites may negatively affect plant growth **(**Fig. 9**).**

Gene expression analysis revealed that only *OsAPX1* genes showed a positive correlation with Mg^2+^ content, while *OsPINI* and *OsCATA* genes showed a positive correlation with jasmonic acid (JA) content. Moreover, *OsSOS* gene expression was found to be negatively correlated with shoot length and root length. Overall, our findings suggest that gene expression is not significantly correlated with plant growth. Flavonoid content, on the other hand, showed a positive correlation with Na^+^ and SA contents, and *OsSOS* genes, thus emphasizing their role in stress response mechanisms. However, no significant positive correlation of polyphenol content was observed with any of the studied parameters except shoot length where it shows positive correlation. Furthermore, no significant correlation was observed between abscisic acid (ABA) with any of the other studied plant parameters. Interestingly, K^+^ showed a positive correlation with Na^+^, indicating that both Na and Ka concentration increased during salt stress.

## Discussion

Salinity is a severe abiotic stress that limits rice production worldwide. The reduction of rice yield under salinity stress depends on salt concentration and duration of exposure to salt stress [29]. High salinity reduces seed germination, plant growth and yield owing to increased Na^+^ uptake, ROS generation, increased endogenous ABA levels, and reduced K^+^ uptake and photosynthesis [30]. However, the PGPB can play a crucial role in rice survival under saline conditions [31]. Our results show that salinity stress reduced rice growth in terms of shoot length, root length, leaf width and chlorophyll index and that bacterial inoculation can counteract the impact of salinity on rice growth without any beneficial effect in absence of salinity However, the salinity-relieving effect of bacteria was most evident under the impact of moderate (80 mM NaCl) salinity, where ART-10 seems the most efficient among the three investigated bacterial isolates (Figs. 2 and 3).

Inoculation of rice plants with PGPB can improve growth attributes and enhance plant salt resistance [32]. Inoculation of bacterial isolates (*Brachybacterium* sp., *Staphylococcus* sp., *Bacillus* sp., and *Kocuria* sp.) to different plants such as radish, lettuce, peanut, strawberry, and wheat conferred resistance to salinity stress and increased plant height and other growth parameters [31–34]. Inoculation of PGPB into salinized rice plants helps promote growth attributes by enhancing certain activities such as 1-aminocyclopropane-1-carboxylate (ACC deaminase) activities, indole-3-acetic acid (IAA) hormones, Phosphate Solubilizing Bacteria (PSB), production of antioxidants, and accumulation of endogenous hormones [35].

Five *OsNHX* genes play a significant role in the adjustment of Na^+^ and K^+^ levels in the rice cytoplasm [15, 36]. *OsNHX* family genes are regulated in inoculated and non-inoculated rice plants, and *OsNHX1* overexpression confers resistance to salinity stress in transgenic rice [15, 37]. *OsNHX1* plays a fundamental role in salinity resistance and can suppress Na^+^ and Li^+^ accumulation in cells. *OsNHX1* is differentially expressed in different plant parts [15]. Our results show that the *OsNHX1* expression was upregulated under moderate salinity stress of 80 mM NaCl but downregulated under high salinity of 160 mM NaCl. Beside this, inoculation with bacterial isolates non-significantly downregulated the gene expression in control plants but upregulated it under salinity stress (Fig. 4A).There are eight *APX* genes in rice; their role is to destroy H_2_O_2_ [38, 39]. Researchers reported the expression of individual *APX* genes in rice plants under salinity stress [40, 41]; for example, *OsAPX* expression has been reported to be enhanced by NaCl stress [42, 43]. The findings of the present study contradict that of previous studies; our findings show that under moderate salinity stress of 80 mM NaCl *OsAPX1* was not expressed while under 160 mM it was reduced. It can be claimed that bacterial inoculation can downregulate *OsAPX1* expression in non-salinized plants and under the impact of moderate salinity but can upregulate it under the impact of high salinity (Fig. 4B). This difference may be due to the use of different rice cultivars. The production of IAA by PGPB is a key feature under salinity stress that enhances plant growth and development [44]. The selected bacterial isolates are known to produce IAA. There are 12 *OsPIN* genes in rice that play a key role under salinity stress by facilitating auxin efflux [45]. *OsPIN* helps in transport of IAA through the cell-to-cell auxin transport mechanism [46], and it is highly regulated under salinity stress [47]. In this study, salinity stress downregulated the expression of *OsPIN1* while bacterial inoculation counteracted the impact of salinity, thus upregulating gene expression but with greater extent in non-salinized and moderately salinized plants relative to highly salinized plants. (Fig. 4C). It has been claimed that bacterial inoculation can enhance expression of *OsPIN2* in *Solanum lycopersicum* [32].

Enzymatic antioxidants such as CAT facilitate the conversion of H_2_O_2_ into O_2_ in living systems [48]. *OsCATA* expression has been reported to be enhanced in bacteria-inoculated plants under salinity stress [32]. Our study shows that salinity stress increased the expression of *OsCATA* in non-inoculated and ART-1-inoculated plants but reduced it in the ART-10-and CAL-8-inoculated plants. Furthermore, bacterial inoculation enhanced the expression of *OsCATA* in rice leaves, particularly in control and moderately salinized plants Fig. 4D). This is comparable to a report showing that salinity stress increases *OsCATA* expression [48]. Plant exposure to salinity stress results in competition of Na^+^ with K^+^ upon uptake; leading to the accumulation of Na^+^ to high intracellular levels that are toxic to enzyme functions [49]. Plants depend on Na^+^ compartmentalization at the cellular and tissue levels and Na^+^ exclusion to overcome the effects of Na^+^ stress [49]. The salt overly sensitive (SOS) salt-tolerant pathway helps expel Na^+^ from cells and maintains an optimal cytosolic Na^+^/K^+^ level in the cell. The upregulation of *OsSOS* under salinity stress [36] coincides with findings of the current study that *OsSOS* is upregulated under salinity stress, but its expression seems to be downregulated by bacterial inoculation (Fig. 4E). The downregulation of *OsSOS* in bacteria-inoculated plants under NaCl stress may be due to the production of antioxidants by bacteria. High Na^+^ accumulation can be toxic and affects normal cell functions, while K^+^ accumulation is vital for the normal functioning of cells. Both Na^+^ and K^+^ are present in the saline environment, but only K^+^ helps maintain the electrolyte and osmotic balance in cells [50]. The Na^+^/K^+^ ratio must be maintained for normal cell functioning. Increasing intracellular K^+^ content reduces Na^+^ absorption and maintains a favorable ratio of Na^+^/K^+^. However, in our study, we observed a deviation from the normal results, as both sodium (Na^+^) and potassium (K^+^) levels were found to be elevated under salt stress conditions. Additionally, we found a positive correlation between the Na^+^/K^+^ratio, as depicted in Fig. 5A and C. Similar findings were reported by [6] where both Na^+^ and K^+^ concentrations were observed to increase during salt stress in the shoots compared to the roots of rice plants. Moreover, a study mentioned in the reference indicated that transgenic and wild plants exhibited contrasting patterns in Na^+^ and K^+^ levels under salt stress [51]. Furthermore, in another investigation, the overexpression of *OsHAK1* gene resulted in a significant increase in K content and Na^+^/K^+^ratio in both roots and shoots, especially under low K supply conditions [52] These results highlight the influence of various genes and different rice cultivars on the Na^+^/K^+^ratio in different parts of the plant. However, it is noteworthy that bacterial inoculation has the potential to counteract the effects of salinity, leading to a decrease in the Na^+^/K^+^ratio compared to non-inoculated plants, as previously reported [32]. Bacteria-inoculated plants under salt stress have an increased and decreased amount of Na^+^ and K^+^, respectively [32]. This correlates with our results showing that the inoculation of bacterial isolates helped maintain the Na^+^/K^+^ ratio within the cell (Fig. 5A, C). Mg^2+^ is an essential macronutrient crucial for photosynthesis because it is the central atom in the tetrapyrrole ring of chlorophyll [51] *a* and *b*; these pigments are essential for light absorption during photosynthesis [53, 54]. Previous studies reported the effects of decreased Mg^2+^ contents on photosynthesis [53, 55]. Our results show that bacterial inoculation can improve Mg^2+^ nutrition of rice plants (Fig. 5B), which agrees with previous studies showing that plants inoculated with bacteria have high Mg^2+^ content [32].

Salinity stress in rice causes the accumulation of ROS, which can cause cell death. Histochemical analysis (using trypan blue staining) is used to monitor cell death under salinity stress [56]. Our results reveal accelerated cell death under the impact of salinity stress, with a relieving effect of bacterial inoculation, especially with CAL-8 (Fig. 6). These findings agree with previous studies showing that inoculating plants with bacteria reduces cell death [56]. In response to abiotic stress, plants produce secondary metabolites to mitigate oxidative stress. Superoxide dismutase (SOD) is a metalloprotein that catabolizes O_2_ to H_2_O_2_ and controls ROS levels in plants under salinity stress [4, 57]. In our study, bacterial inoculation enhanced SOD activity of salt-stressed rice leaves (Fig. 7A). Similar results were reported using different bacterial isolates to mitigate the impact of salinity [32]. DPPH radical scavenging activity is a measure of non-enzymatic antioxidant activity. In the present work, although the effect of bacterial inoculation in increasing DPPH-scavenging activity is appreciable, yet it is statistically non-significant (Fig. 7B). The role of bacterial inoculation in mitigating the impact of abiotic stress via enhancement of DPPH scavenging activity of higher plants has been reported [13]. Plants also produce secondary metabolites (including flavonoids and polyphenols) to reduce oxidative stress under stress conditions [13]. Polyphenols and other secondary metabolites are biosynthesized via the phenylpropanoid pathway [13]. Polyphenols act as antioxidants by scavenging free radicals, and their activity depends on the number and position of the hydroxyl substituents. They terminate the propagation of the free radical chain reaction by the binding of a hydrogen atom of the polyphenol with the free radical [58]. This study shows that the polyphenol content of rice leaves was non-significantly increased under salinity stress, with marginal interaction from bacterial inoculation except with the significant reduction observed upon inoculation with ART-10 (Fig. 7C). Flavonoids play a crucial role in photosynthesis as catalysts and help in phosphorylation by regulating ion channels [59]. In our results, flavonoid content increased under salinity stress in non-inoculated plants; however, the application of different bacterial isolates reduced flavonoid content of salinized plants but increased it in the control non-salinized plants (Fig. 7D). These results show that inoculating bacterial isolates under salinity stress may be involved in the regulation of antioxidant mechanisms that mitigate the negative effects of environmental stress. Phytohormones are chemicals produced inside the plant body and play a key role in mitigating stress by enhancing plant growth and development [60]. Salicylic acid is a stress- hormone crucial in plant defense against abiotic stresses [61]. In the present work, endogenous SA content of rice leaves was significantly increased under salinity stress, with a peak at 80 mM NaCl and marked salinity × bacteria interaction. Bacterial inoculation can increase SA content of leaves in non-stressed as well as the highly salinized plants, but it led to considerable reduction in the moderately salinized plants (Fig. 8A). In agreement with the present results, inoculation of salinity stressed *Solanum lycopersicum* plants with PGPB enhanced endogenous SA levels [32]. Abscisic acid is another phytohormone that accumulates in plants under abiotic stress conditions [62]. Many studies have documented the mitigating effect of endogenous ABA under abiotic stress conditions [62]. In our study, ABA content of rice leaves was subjected to significant salinity × bacteria interaction. Whereas salinity stress induced an increase in ABA content of rice leaves in non-inoculated it caused a decrease in inoculated plants. Likewise, whereas bacterial inoculation increased ABA content of leaves in non-salinized plants it led to a reduction under the impact of salinity (Fig. 8B). Similar to our findings, salinity stress increased ABA content in soybean plants, whereas bacterial inoculation reduced it [63, 64]. Jasmonic acid, another signaling molecule, can mitigate the impact of stress via alleviating oxidative stress damage [65]. Salinity stress increased endogenous JA levels in *Arabidopsis thaliana* [66] and rice [67]. Various PGPB have been used to mitigate the impact of abiotic stress through reprogramming JA biosynthesis [64, 68]. In our study, the effect of salinity in increasing JA content of rice leaves was evident only in non-inoculated plants, whereas the increase due to bacterial inoculation was considerable and consistent at all salinity levels (Fig. 8C).

## Conclusion

In conclusion, salt stress severely undermines rice production by reducing yield, plant growth, and seed germination. However, using Plant Growth Promoting Bacteria (PGPB) has the potential to mitigate salinity’s negative effects on rice plants. The inoculation of PGPB improves salt resistance, stress tolerance, and growth characteristics. Numerous factors, including shoot length, root length, leaf width, chlorophyll index, and nutrient uptake, can be improved by bacterial isolates. Furthermore, PGPB inoculation regulates the expression of important genes including *OsNHX1*, *OsAPX1*, *OsPIN1*, and *OsCATA* that are involved in ion homeostasis and antioxidant defense. Additionally, the application of PGPB assists in maintaining a proper Na+/K + ratio, which is essential for cellular function when exposed to salinity stress. Additionally, bacterial inoculation minimizes cell death and boosts the activity of enzymes which combat free radicals, such superoxide dismutase (SOD). Overall, PGPB inoculation has the potential to increase rice’s tolerance to salinity stress and increase crop growth in salt stress.

## Experimental procedures

### Isolation, screening and identification of bacterial endophytes

The endophytic bacteria used in this work were isolated from the rhizosphere of two halophytes: *Artemisia princeps* and *Chenopodium ficifolium* from Pohang Beach (South Korea). Bacterial isolates were characterized in the Crop Physiology Laboratory, Department of Applied Biosciences, Kyungpook National University, Korea [2, 69]. These strains were identified by sequencing partial 16 S ribosomal RNA (rRNA) [70]. Total DNA was isolated, and the 16 S rRNA gene was PCR-amplified using the 27 F primer (5′-AGAGTTTGATC(AC)TGGCTCAG-3′) and 1492R primer (5′-CGG(CT)TACCTTGTTACGACTT-3′), complementary to the 5′ and 3′ ends of the prokaryotic 16 S rRNA, respectively. The BLAST search program NCBI and EzTaxon-e were used to identify the homology of the different nucleotide sequences of the selected endophytic bacterial strains. The most similar sequences with the highest homology were used to construct a phylogenetic tree using the neighbor-joining method in MEGA software version 6.0 and were aligned at 1,000 bootstrap replications.

### Experimental design for salinity-bacteria interaction on rice growth and performance

Seeds of rice (*Oryza sativa* L.) cv. “Ilmi” were provided by the Plant Molecular Breeding Laboratory, Kyungpook National University, Korea. The seeds were disinfected with 500 µL/L Spotak pesticide (Hankooksamgong, Seoul, South Korea and soaked in water at 34 °C in an incubator. After three days, the pre-germinated seeds were transferred to the specialized soil (Doobaena plus) (raw material name and ratio %: Cocopeat 27, peatmoss 10, vermiculite 34, Masato 10, diatomite 13, bara mesh 5.5, fertilizer 0.48, and humectant 0.2.) provided by Nongkyung Co. Ltd, Korea, until successful growth. The experiment is factorial with two main factors and three replications, each was five plants, in a completely randomized design. The whole experiment consists of three groups; Control, 80 mM NaCl stress and 160 mM NaCl stress following published experimental design with slight changes [71]. Each group has four levels of inoculation: non-inoculated (NI), inoculation with ART-1, inoculation with ART-10 and inoculation with CAL-8.

Bacterial isolates, grown on LB medium, were centrifuged at 6,000 × g for 10 min at 4 °C. The pellets were suspended in distilled water, and the pots were inoculated with freshly prepared bacterial culture for 15 days with every 5 days interval. Salinity stress (80 mM and 160 mM NaCl) was applied to each pot three times at five days intervals following the 15-day treatment with the bacterial isolate. Morphological measurements (shoot length, root length leaf width and chlorophyll index) were measured after 15 days of salinity treatment. Leaf samples were collected for analyses, including enzymatic and non-enzymatic antioxidant activities, quantification of endogenous phytohormones, ion content and gene expression.

### Measurement of growth and chlorophyll index

Plant growth was estimated in terms of shoot length, root length, and leaf width. Chlorophyll index was measured using a portable chlorophyll meter (SPAD 502; Konica Minolta, Japan). Measurements were taken at three points for each leaf: leaf tip, middle leaf, and leaf base and averaged for the chlorophyll index of leaf.

### Relative gene expression using RT-PCR

RNA was extracted from rice leaves using the RNeasy plant mini kit (Qiagen, Hilden, Germany) according to the manufacturer’s instructions, whereas the UltraScript 2.0 cDNA synthesis kit was used for cDNA synthesis. qRT-PCR was performed using a qPCRBIO SYBR Green kit on an Eco Real-Time (Illumina, Singapore) machine; *OsActin* was used as the reference gene. The expression of several genes was measured in triplicate (Table 1**)**.

**Table 1:** Primers and accession numbers of selected genes for qRT-PCR.

Gene	Forward primer	Reverse primer	Accession No
*OsACT1*	TGAATCTGGTCCAGGCATCG	TGGGACGCATGCAAACAATC	XM 015785964.2
*OsNHX1*	AGCGGCATTCTCACCGTATT	GAGCAATCGACACAGCTCCT	XM_015789089
*OsAPX1*	GGGTTCTGACCACCTAAGGC	TGGCCATAGGCCGAACAAAT	AK061841
*OSPIN1A*	GACAGGGAGGACTACGTGGA	GAGGCTGGAGTAGGTGTTCG	NM_001401976.1
*OsCATA*	CAAACCCCTCTCACTCCCAG	TCGCGGGTGTAGAACTTGAC	NM_001401748
*OsSOS1*	GGCAGGATAATGTGGTGCTT	TGAGCAGCAGGCAATATCAC	AY785147

### Quantification of endogenous phytohormones

Endogenous ABA, SA and JA were quantified in plant leaves. Abscisic acid assay was performed by grinding the leaves into fine powder and mixing 3 mg of powder with 30 ml extraction solution (95% isopropanol and 5% glacial acetic acid) and 10 ng of ABA standard ([±]-3,5,5,7,7,7-d^6^) [72]. The extract was dried and methylated with diazomethane. Abscisic acid was quantified in triplicates using GC-MS/SIM (5973 Network mass selective detector and 6890 N Network GC system: Agilent Technologies, Palo Alto, CA, USA). Salicylic acid (SA) was quantified using the protocol adopted by Jan et al. [73]. Briefly, Leaf samples were freeze-dried, and the extract of 0.2 g was fractionated by high-performance liquid chromatography (HPLC), and SA was quantified using fluorescence detection. Endogenous JA was quantified using the method adopted by Baldwin et al. 1997 [74]. Briefly, 0.3 g of frozen leaves was ground in liquid nitrogen and treated with an extraction solution (10 mL of 70% acetone, 30% 50 mM citric acid (v/v) and 50 ng of JA standard (9, [_10−2_H_2_]-9,10-dihydro-JA). The extract was kept at low temperature overnight to evaporate the highly volatile organic solvents and retain the less volatile fatty acids. The remaining aqueous solution was filtered and extracted three times using 30 ml diethyl ether. A solid-phase cartridge was used for extract loading, and the cartridge was washed twice with 5 ml of trichloromethane/ 2-propanol mixture (2:1, v/v). The standard and bound JA were washed with 1 ml of diethyl ether/acetic acid mixture (98:2, v/v). The samples were then fractionated using GCMS (6890 N network GC system). Fragment ions were analyzed using the following parameters: m/z = 83 AMU, relative to the base peaks of JA and [9,10-2H2]-9,10-dihydro-JA.

### Estimation of antioxidant activities

The superoxide dismutase (SOD) and DPPH scavenging activities as well as the content of total flavonoids (TFC), and total phenolics (TPC), were assayed in rice leaves. Leaf samples were ground into a fine powder, then homogenized in 50 mM phosphate buffer (pH 7.5) containing 1% (w/v) polyvinylpyrrolidone (PVP), 0.1 mM EDTA, and 0.5% (w/v) Triton X-100. Superoxide dismutase activity was measured spectrophotometrically (Multiskan GO, Thermo Fisher Scientific, Waltham, MA, United States) at 560 nm using the method adopted by Marklund et al. [75]. The DPPH scavenging activity was assayed to monitor the free radical scavenging activity of plant leaves [76]. Briefly, leaf samples were extracted in MeOH, and a reaction mixture containing 5 mg DPPH in 50 ml MeOH was prepared. The reaction mixture and the methanolic leaf extract (1:1) were kept in the dark at room temperature for 30 min, and absorbance of the samples was measured at 517 nm. The radical scavenging activity was expressed as percentage lowering in absorbance below the standard DPPH solution. Total polyphenol content was measured using the Folin-Ciocalteu colorimetric method [77]. Briefly, leaf samples were extracted using 80% MeOH, and 50 µl of the extract were added to a mixture of 1 ml 2% Na_2_CO_3_ and 50 µl of 1 N Folin-Ciocalteu reagent. The reaction mixture was kept at room temperature for 30 min, and absorbance was measured at 750 nm. Total phenolic content was calculated as mg gallic acid equivalent per gram extract with reference to a standard curve of gallic acid in the range of 750 nm. Quantitative measurements were performed based on a standard calibration curve of six points: 20, 100, 200, 300, 400, 500 mg/l of gallic acid. The total flavonoid content was measured according to the procedure of Zhishen et al. [78]. Briefly, leaf samples were extracted with 80% MeOH. An aliquot (100 µl) of the methanolic extract were mixed with a reaction mixture containing 500 µl MeOH, 50 µl of 1 M NaOH, 50 µl of 10% AlCl_3,_ and 300 µl distilled water and incubated at room temperature for 30 min. The absorbance was measured at 510 nm and flavonoid content of leaves was calculated as mg quercetin equivalent per gram leaf with reference to a standard curve of quercein in the range of 500 nm. Quercetin was used to make the calibration curve. 10 mg of quercetin was dissolved in ethanol 96% and diluted to 2, 4, 6, 8 and 10 µg/mL.

### Histochemical analysis

The hypersensitivity response of treated plants was measured following the procedure of Yin et al. [79]. Cell death was measured by using the trypan blue staining. Briefly, leaves were placed in test tubes containing trypan blue staining solution (2.5 mg of trypan blue per ml, 25% [wt/v] lactic acid, 23% water-saturated phenol, 25% glycerol, and H_2_O), boiled for 10 min, and kept in the dark for 12 h. Next, the leaves were treated with 25 mg/ml chloral hydrate solution for 24 h to remove leaf color, and blue spots on the leaves were recorded and photographed.

### Inductively coupled plasma mass spectrometry (ICP-MS)

Inductively coupled plasma mass spectrometry was used to quantify Na^+^, K^+^ and Mg^2+^ in leaves. Fresh leaf samples (0.2 g) were crushed to a fine powder in liquid nitrogen, extracted in a mixture containing 7 ml of 65% HNO_3_ and 1 ml of 30% H_2_O_2_, kept in a microwave at 180 ℃ for 20 min, and cooled for 40 min [81]. The mineral content of the extract was quantified using ICP-MS (9ICP = MS; Optima 7900DV, Perkin-Elmer, Waltham, MA, USA).

#### Confirmation of bacterial isolates in inoculated plants

For confirmation of bacterial isolates, we washed the roots of control and inoculated plants to remove the soil. After that roots were grind in liquid nitrogen using mortar and pestle. Total DNA was isolated, and the 16 S rRNA gene was PCR-amplified using the 27 F primer (5′-AGAGTTTGATC(AC)TGGCTCAG-3′) and 1492R primer (5′-CGG(CT)TACCTTGTTACGACTT-3′), complementary to the 5′ and 3′ ends of the prokaryotic 16 S rRNA, respectively. The BLAST search program NCBI and EzTaxon-e were used to confirm the presence of selected bacterial isolates in inoculated plants.

### Statistical analysis

The correlation between the studied traits was evaluated in terms of Pearson’s correlation coefficient by using GraphPad Prism software (version 5.01, GraphPad, San Deigo, CA, USA).

## Data Availability

These sequence data have been submitted to the GenBank database: OP999377 (ART-1), OP999612 (ART-10), and OP999615 (CAL-8). The address is as follows: http://www.ncbi.nlm.nih.gov.
